# Pyogenic Granuloma in a One-Year-Old Child: A Rare Entity

**DOI:** 10.7759/cureus.55487

**Published:** 2024-03-04

**Authors:** Jeswin M Thomas, Subbalekshmi T, Joisy P Jame, Tibin Baby, Miranda A George, Anandhukrishnan E

**Affiliations:** 1 Department of Pediatrics and Preventive Dentistry, Pushpagiri College of Dental Sciences, Kerala University of Health Sciences, Thiruvulla, IND; 2 Department of Oral and Maxillofacial Pathology, Pushpagiri College of Dental Sciences, Kerala University of Health Sciences, Thiruvulla, IND

**Keywords:** pediatric, local trauma, surgical excision, gingival lesion, pyogenic granuloma

## Abstract

Pyogenic granuloma (PG) is a benign vascular neoplasm seen in the first and second decades of life, and it has a female predilection. It presents as a small reddish exophytic lesion, gingiva being the most common site. This article describes an unusual presentation of PG in a one-year-old female child and highlights the importance of its early diagnosis and management to avoid discomfort and distress in these patients. The diagnosis was verified by histological examination, which revealed significant markers such as endothelial growth, vascular abundance, and chronic inflammatory cell infiltration. The chosen treatment protocol was surgical excision, which led to a successful outcome with no symptoms of recurrence, as confirmed by thorough follow-up examinations.

## Introduction

Pyogenic granuloma (PG), also known as lobular capillary hemangioma or granuloma pyogenicum [1], is a benign vascular tumor that commonly arises in the oral cavity. PG is commonly seen in young children and pregnant women [2]. It predominantly occurs in the first and second decades of life in young females, with a male-to-female ratio of 1:99; the size of the lesion varies in diameter from a few millimeters to several centimeters but rarely exceeds 2.5 cm [2,3]. Histologically, PG is often described as a "hemangiomatous granuloma" due to the profusion of blood vessels and the inflammatory nature of the lesion. Another synonymous term, "granuloma telangiectacticum," reflects the lesion's diverse coloration, ranging from shades of red to pink.

Clinically, PG typically starts as a modest, slowly-growing exophytic growth with a smooth or lobulated surface. It manifests as an erythematous papule, often radiating shades of red, and may feature a pedunculated or sessile base [4]. Although most PG lesions are confined to the gingiva, significant bone loss can occur in rare cases. While PG may affect individuals of all ages, it is most commonly seen in the second decade of life, with a pronounced female predilection often attributed to hormonal fluctuations.

Studies have described cases of Kaposi's sarcoma, which has symptoms similar to PG, closely mimicking PG [5,6,7], and delved into treatment strategies for PG, highlighting surgical excision as the gold standard while also considering recurrence rates and associated factors [8,9]. This report describes a rare occurrence of PG in a one-year-old child and emphasizes the importance of early intervention in these patients.

## Case presentation

**Figure 1:**
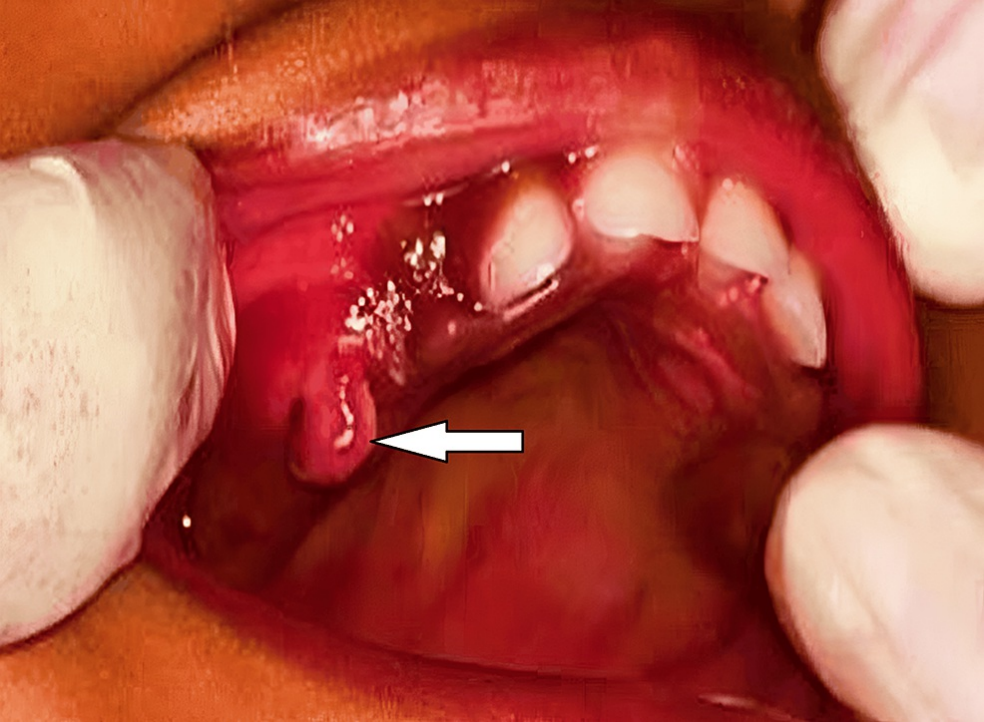
Preoperative image of the pyogenic granuloma The arrow shows granuloma intraorally

A one-year-old female child was referred to the Department of Pediatric and Preventive Dentistry with a chief complaint of growth on the buccal aspect of the upper right back primary first molar region. The parent had initially noticed the growth a month back. They stated that the growth had been bleeding for two days. On intraoral examination, a single pedunculated gingival growth measuring 0.5 x 0.5 x 0.3 cm, oval in shape, greyish-white in color, and soft in consistency was identified on the maxillary right primary molar region (Figure 1).

The surgical removal of the lesion was planned based on clinical evaluation. Given the minor's status, the treatment approach and expected results were thoroughly discussed with the legal guardian. The consent for surgery was obtained from the patient's legal guardian as per ethical and medical standards. The child being in the precooperative age group, physical restraints were used while performing the procedure.

**Figure 2:**
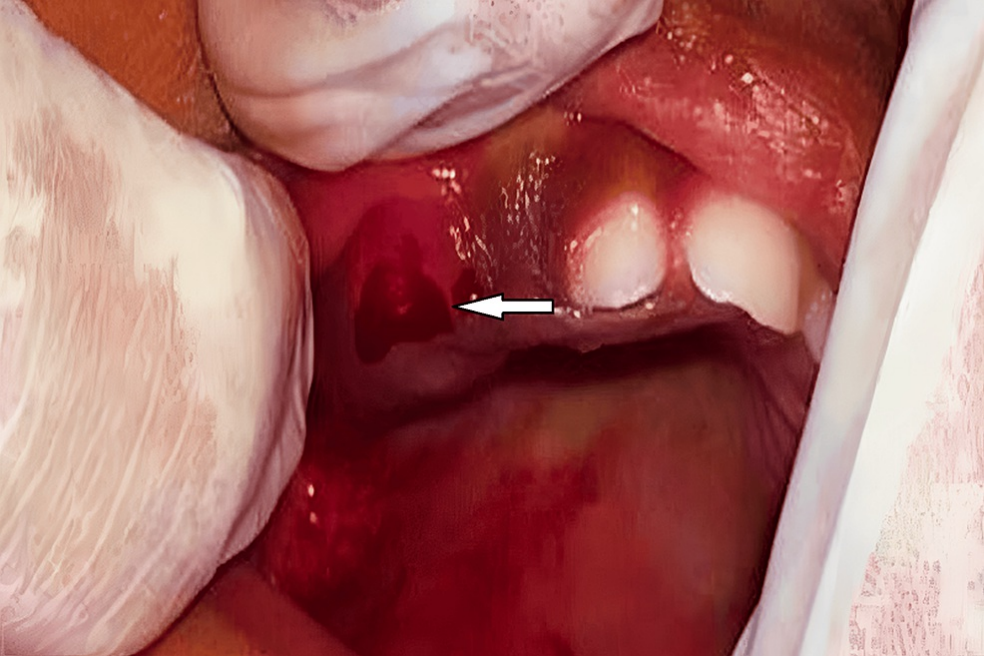
Intraoperative image of the excision of the granuloma The arrow shows the incision placed

**Figure 3:**
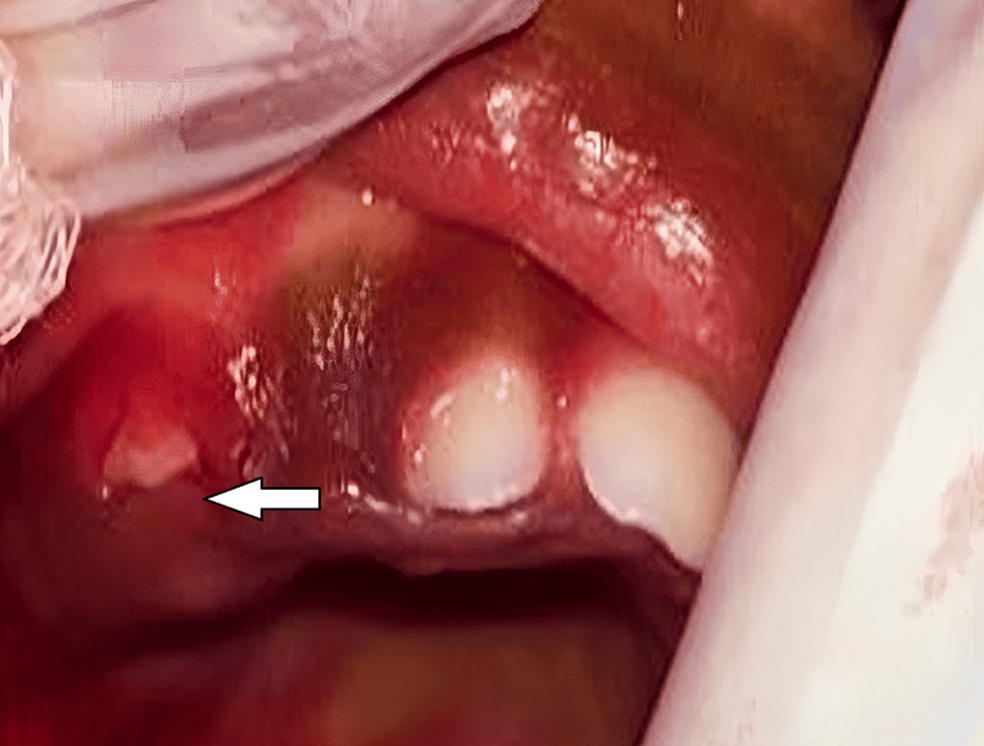
Immediate postoperative image after the excision of the granuloma The arrow shows the tooth exposed just after the incision

A thorough hematological evaluation of the patient was performed before the removal of the lesion. Bleeding time, clotting time, hemoglobin, and total and differential leukocyte counts were all found to be within normal limits, confirming that the patient was ready for surgery. The lesion was surgically excised under local anesthesia. Lignocaine hydrochloride gel 2% was topically applied on the lesion and its periphery followed by infiltration with 1 ml local anesthesia (lox 2% adrenaline 1:200000). The growth was held with Adson tissue forceps and the incision was made with a scalpel of size number 11 on the buccal aspect of gingiva about the right maxillary primary molar region. No sutures were placed as hemostasis was achieved by a gauze pack. Analgesics were prescribed. Subsequently, the excised specimen was sent for a comprehensive histopathological analysis. The patient was recalled after two weeks for a follow-up (Figures 2, 3).

**Figure 4:**
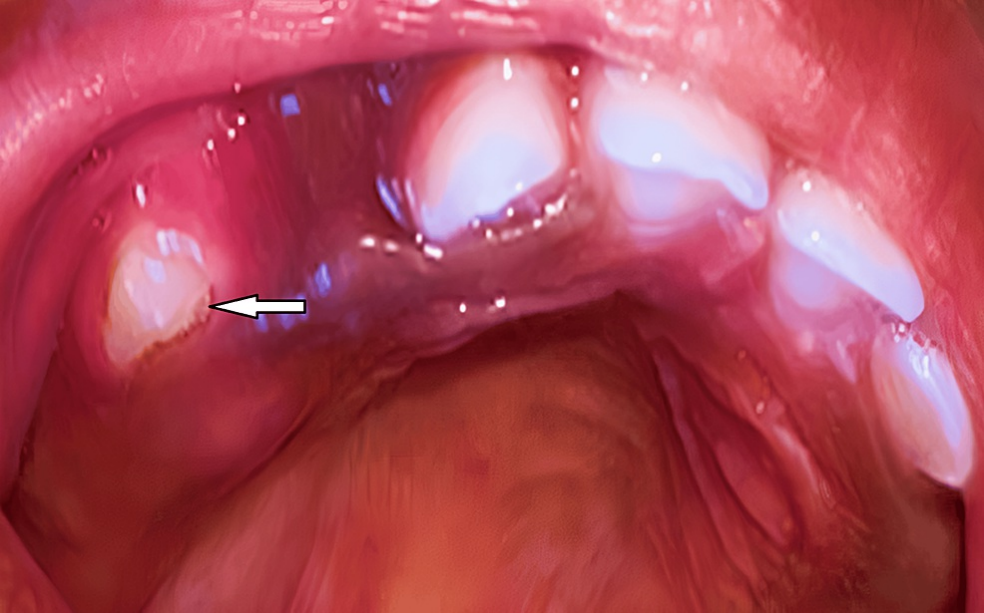
Postoperative image at the follow-up The arrow shows the tooth after healing

To ensure the patient's postoperative well-being and monitor for any potential recurrence, follow-up examinations were conducted at two weeks and six months post-surgery (Figure 4).

**Figure 5:**
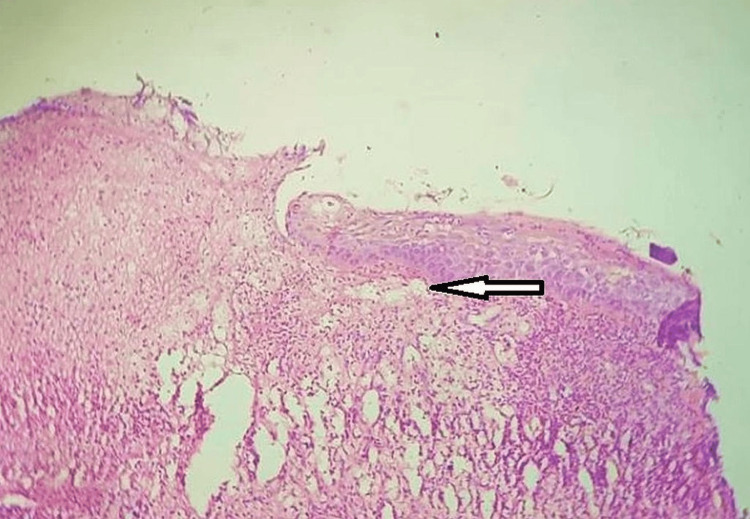
Histopathological section image of the tissue showing the endothelium-lined arterial channels The arrow shows endothelium-lined blood channels

These follow-up assessments served to confirm uneventful healing and to detect any early signs of recurrence, enabling prompt intervention if necessary. Histological analysis revealed a discontinuous, thin nonkeratinized stratified squamous epithelium, indicating the origin of the tissue within the oral cavity. Notably, the underlying connective tissue included a plethora of endothelium-lined arterial channels, emphasizing the vascularity of the lesion. Further investigations indicated that endothelial cells and fibroblasts were actively proliferating, with budding capillaries adding to the intricate vascular network. In addition, there was an intensive and noticeable chronic inflammatory cell infiltration into the tissue, indicating a continuous inflammatory response within the lesion's microenvironment. These histopathological findings contributed to a full understanding of the nature and cellular composition of the lesion, indicating PG (Figure 5).

## Discussion

PG is an inflammatory hyperplasia that occurs commonly in the oral cavity and less frequently in the digestive tract; it also affects the skin in some cases. We presented the case of a one-year-old female child with PG, a very unusual entity. While PG is associated with diverse etiological factors, including chronic trauma, cyclosporine use, and occlusal interferences [10-17], this case was notably attributed to local trauma that could have occurred during tooth-brushing, as seen in the case report by Gera et al. (2023) [18]. Histological examination of the excised tissue revealed characteristic features consistent with PG, such as discontinuous thin, nonkeratinized stratified squamous epithelium and a profusion of endothelium-lined vascular channels. The proliferation of endothelial cells and fibroblasts, as well as budding capillaries, further corroborated the diagnosis. Significantly, an intense chronic inflammatory cell infiltration was observed, highlighting the inflammatory nature of this lesion, in line with a previous study by Radia et al. in 2018 [19].

PG is most commonly encountered in the first and second decades of life, with a notable female predilection; the male-to-female ratio is reported to be 1:99 [3]. Its occurrence in pregnant women, often referred to as "pregnancy tumor" or "pregnancy epulis," is well-documented and is attributed to hormonal fluctuations during pregnancy [4]. While it predominantly manifests in the oral cavity, particularly the gingiva, PG can also occur in other mucous membranes and skin, emphasizing its diverse clinical presentation [1]. The exact etiology of PG is multifactorial and not fully understood. Trauma, such as that caused by chronic irritation, dental procedures, or ill-fitting appliances, is a common precipitating factor [2]. Hormonal influences, as seen in pregnancy or with the use of contraceptives, may also contribute to its development [5]. Additionally, vascular endothelial growth factors (VEGF) and angiogenic factors play a crucial role in the pathogenesis of PG, by promoting the formation of abnormal blood vessels and the characteristic vascular proliferation seen histologically [6].

The primary treatment for PG involves surgical excision, which is considered the gold standard for its management. The surgical approach depends on the lesion's size and location, and the goal is to completely remove the lesion. Excision should be followed by careful histopathological examination to confirm the diagnosis and rule out any underlying malignancy. Alternative treatment modalities, such as cryosurgery, laser therapy, sclerotherapy, or corticosteroid injections, may be considered for smaller lesions or cases where surgery is less feasible [9]. Recurrence rates for PG vary but are relatively common, particularly in gingival lesions. Factors contributing to recurrence include incomplete excision, persistent irritants, and hormonal influences. Close follow-up is essential to monitor for recurrence and ensure proper wound healing [10].

Treatment options for PG vary depending on the lesion's size, and in our case, surgical excision was chosen as the most suitable approach, based on recommendations in the literature. While alternative treatments like cryosurgery, laser therapy, sclerotherapy, and corticosteroid or ethanol injections exist, the choice of treatment should be tailored to the individual case [4]. The child's parent was instructed to refrain from feeding the patient hot food and provide a soft diet for 24 hours. Analgesics was prescribed for three days. Recurrence remains a problem with PG. This can happen due to several reasons, including the failure to excise the tissues that caused the lesion, repeated trauma by brushing or food impaction, or bad oral hygiene. Hence, close follow-ups of these patients are essential, especially in cases involving gingival PG, given their higher recurrence rate, as per the studies by Vilmann et al. [20] and others [21-23]. In our case, meticulous monitoring over six months revealed no signs of recurrence, demonstrating the effectiveness of the chosen treatment approach and the importance of vigilant post-treatment care.

## Conclusions

PG can be particularly distressing, especially when it afflicts very young children. Left untreated, this condition can escalate in size, leading to significant pain and discomfort. Given its relative rarity in children, the importance of early intervention and appropriate treatment cannot be overstated. Addressing PG promptly not only relieves the child's discomfort but also substantially improves the overall quality of life. Early diagnosis and management are critical in ensuring that the child has a more comfortable and healthier life.

## References

[1] Giblin AV, Clover AJ, Athanassopoulos A, Budny PG (2007). Pyogenic granuloma - the quest for optimum treatment: audit of treatment of 408 cases. J Plast Reconstr Aesthet Surg.

[2] Neville BW, Damm DD, Allen CM, Bouquot JE (2002). Oral & Maxillofacial Pathology. W B Saunders.

[3] Rowe L (1958). Granuloma pyogenicum: differential diagnosis. AMA Arch Derm.

[4] Regezi JA, Sciubba JJ, Jordan RCK (2003). Oral pathology: clinical pathologic considerations. https://dl.konkur.in/post/Book/Dentistry/Oral-Pathology-Clinical-Pathologic-Correlations-7th-Edition-%5Bkonkur.in%5D.pdf.

[5] (1995). Enzinger and Weiss's Soft Tissue Tumors, 7th Edition. St Louis: Mosby.

[6] Calonje E, Wilson-Jones E (1997). Vascular tumors: tumors and tumor-like conditions of blood vessels and lymphatics. Lever’s Histopathology of the Skin. 8th Edition.

[7] Ryan P, Aarons S, Murray D (2002). Human herpesvirus 8 (HHV-8) detected in two patients with Kaposi's sarcoma-like pyogenic granuloma. J Clin Pathol.

[8] Powell JL, Bailey CL, Coopland AT, Otis CN, Frank JL, Meyer I (1994). Nd:YAG laser excision of a giant gingival pyogenic granuloma of pregnancy. Lasers Surg Med.

[9] White JM, Chaudhry SI, Kudler JJ, Sekandari N, Schoelch ML, Silverman S Jr (1998). Nd:YAG and CO, laser therapy of oral mucosal lesions. J Clin Laser Med Surg.

[10] Nirmala SVSG, Vallepu R, Babu M, Dasarraju RK (2016). Pyogenic granuloma in an 8-year-old boy - a rare case report. J Pediatr Neonatal Care.

[11] Kumar D, Agarwal T (2019). A case report: aggressive pyogenic granuloma. Dent J Adv Stud.

[12] Razi M, Debnath S, Qamar S, Tripathi A (2019). Management of pyogenic granuloma in pediatric patients using electrocautery-case reports. IP Int J Periodontol Implantol.

[13] Verma PK, Srivastava R, Baranwal HC, Chaturvedi TP, Gautam A, Singh A (2012). Pyogenic granuloma-hyperplastic lesion of the gingiva: case reports. Open Dent J.

[14] Cheney-Peters D, Lund TC (2016). Oral pyogenic granuloma after bone marrow transplant in the pediatric/adolescent population: report of 5 cases. J Pediatr Hematol Oncol.

[15] Kanda Y, Arai C, Chizuka A (2000). Pyogenic granuloma of the tongue early after allogeneic bone marrow transplantation for multiple myeloma. Leuk Lymphoma.

[16] Bachmeyer C, Devergie A, Mansouri S, Dubertret L, Aractingi S (1996). Pyogenic granuloma of the tongue in chronic graft versus host disease (Article in French). Ann Dermatol Venereol.

[17] Lee L, Miller PA, Maxymiw WG, Messner HA, Rotstein LE (1994). Intraoral pyogenic granuloma after allogeneic bone marrow transplant. Report of three cases. Oral Surg Oral Med Oral Pathol.

[18] Gera D, Tanwar A, Nigam AG, Jain S, Sharma V (2023). Pyogenic granuloma in a 6-year-old boy - a rare case report. Int J Contemp Pediatr.

[19] Radia H, Oum KE, Cherkaoui A (2018). Pyogenic granuloma of the gingiva: a case report. Int J Contemp Res.

[20] Vilmann A, Vilmann P, Vilmann H (1986). Pyogenic granuloma: evaluation of oral conditions. Br J Oral Maxillofac Surg.

[21] Muthukrishnan K, Gajula SP, Venkatachalamoorthi V, Subramaniam KV (2021). Recurrent episodes of oral pyogenic granuloma at different site in an 8-year-old girl: an unusual presentation. Int J Clin Pediatr Dent.

[22] Neville BW, Damm DD, Allen CM, Angela CC (1995). Oral and Maxillofacial Pathology. Philadelphia, PA: W.B. Saunders.

[23] Jafarzadeh H, Sanatkhani M, Mohtasham N (2006). Oral pyogenic granuloma: a review. J Oral Sci.

